# Iterative deep neural networks based on proximal gradient descent for image restoration

**DOI:** 10.1371/journal.pone.0276373

**Published:** 2022-11-04

**Authors:** Ting Lv, Zhenkuan Pan, Weibo Wei, Guangyu Yang, Jintao Song, Xuqing Wang, Lu Sun, Qian Li, Xiatao Sun

**Affiliations:** 1 College of Computer Science and Technology, Qingdao University, Qingdao, Shandong Province, China; 2 School of Engineering and Applied Science, University of Pennsylvania, Philadelphia, Pennsylvania, United States of America; Shandong Normal University, CHINA

## Abstract

The algorithm unfolding networks with explainability of algorithms and higher efficiency of Deep Neural Networks (DNN) have received considerable attention in solving ill-posed inverse problems. Under the algorithm unfolding network framework, we propose a novel end-to-end iterative deep neural network and its fast network for image restoration. The first one is designed making use of proximal gradient descent algorithm of variational models, which consists of denoiser and reconstruction sub-networks. The second one is its accelerated version with momentum factors. For sub-network of denoiser, we embed the Convolutional Block Attention Module (CBAM) in previous U-Net for adaptive feature refinement. Experiments on image denoising and deblurring demonstrate that competitive performances in quality and efficiency are gained by compared with several state-of-the-art networks for image restoration. Proposed unfolding DNN can be easily extended to solve other similar image restoration tasks, such as image super-resolution, image demosaicking, etc.

## 1 Introduction

Image restoration is an ill-posed inverse problem to recover clean images from degraded images. It can be used in many significant applications, such as medical image processing, face identification, traffic statistics, cultural relics reconstruction, etc.

Mathematically, image restoration problems are defined as *y* = *Hx* + *n*, where y and x are degraded images and clean images, *H* represents degradation matrix, *n* in this paper denotes Additive White Gaussian Noise (AWGN). Different image restoration tasks are expressed by different operations of matrix *H*. For example, when *H* is an identity matrix, image restoration problems are denoising tasks. When *H* is a blurry matrix about 2D convolution operations, they turn into deblurring tasks.

The methods to solve linear inverse problems are divided into two main categories, i.e., model-based methods [1–7] and learning-based methods [8–12]. Variational methods minimize energy functions as tools to solve linear inverse problems. Variational model is expressed as

$$\begin{array}{c} x=\text{arg}\;\underset{x}{\text{min}}\;\{E(x)=D(x,y)+\lambda R(x)\}, \end{array}$$ (1)

where *y* is degraded inputs, and *x* is reconstructed outputs. D denotes a data fidelity term to guarantee that solutions of image restoration accord with degradation process. R is a prior (regularization) term with a regularization parameter *λ* that ensures image features. It is flexible to handle different image tasks by simply integrating different degradation operations (noise level, blur kernel, and downsampling factor) into equations. Whereas, model-based methods lack an intuitive evaluation. Another approach is deep learning with a pre-learned function F(y;Θ), where Θ denotes trainable parameters. Data-driven approaches tend to enjoy better performance. However, learning-based methods suffer from black-box properties and have limitations in specified tasks.

Above two categories of methods have their advantages and disadvantages. Therefore, it would recently be attractive to explore their integration with respective merits, dubbed as unrolling iterative methods. Such an integration results in Plug-and-Play (PnP) methods which replace proximal operators with learning-based denoiser prior. Splitting algorithms of PnP methods split an energy function into multiple stand-alone solution functions. Zhang et al. [13] used Half Quadratic Splitting (HQS) to split a problem into a data recovery term and a feature expression term. Fast Fourier Transform (FFT) solves a data recovery sub-problem due to an analytical solution. The denoiser settles a feature expression sub-problem. Lei et al. [14] put forward that Deep Convolutional Neural Networks (DCNN) are inserted into Split Bregman (SB) methods. Chan et al. [15] proved that plug-and-play Alternating Direction Method of Multipliers (PnP-ADMM) converges to a fixed point for any denoising algorithms satisfying asymptotic criterion. Methods without splitting algorithms open a new door to integrate degraded operations into equations. Al-Shabili et al. [16] utilized Bregman Proximal Gradient Methods of PnP (PnP-BPGM) to reduce splitting algorithms for solutions of Poisson inverse problems. Gavaskar et al. [17] proposed that plug-and-play Fast Iterative Shrinkage/Thresholding Algorithm (PnP-FISTA) is achieved in virtue of Asymmetric denoisers. Nair et al. [18] analyzed the PnP convergence of Iterative Shrinkage/Thresholding Algorithm (ISTA) using asymmetric denoisers. Although superior performances through pre-training can be harvested by PnP approaches, several conceptual problems remain to be addressed. First, hand-crafted parameter adjustment significantly affects the time costs. Second, dynamic characteristics of model optimization are ignored by fixed parameters. Dynamic process to find a better solution is not represented by constant parameters. Third, it is difficult to know which parameters are optimal, and, finally, soundness of image reconstruction profoundly interferes with fluctuation of parameters.

To address above drawbacks, we advocate an end-to-end training structure with trainable parameters to unroll iterative algorithms. It not only infers desirable high-quality images or missing high-frequency information from a large number of degraded images, but also adjusts given parameters to learn automatically. Dong et al. [19] used deep unfolding networks to make up for the insufficiency of parameter tuning. Liu et al. [20] unrolled ADMM into a proximal alternating direction network and used dynamic parameters to guarantee at least fixed-point convergence when dealing with unknown and intractable regularization terms. Yang et al. [21] put forward that unrolling ADMM networks realize discriminative learning from training data instead of setting hyperparameters by hand in traditional compressive sensing methods. Aimed at artificial tweaking of PnP methods, Wei et al. [22] proposed a parameter automatic tuning network to achieve automatically tuning of internal parameters, which is a tuning-free PnP proximal algorithm. Undoubtedly, computational costs by hand can be greatly controlled by a self-learning technique of parameters.

The contributions of this work are outlined below:

- The proximal gradient descent algorithm is unfolded into a novel and simple Iterative Deep Neural Network (IDNN) with the U-Net denoiser. Attention mechanism incorporated into the denoisers effectively understands which image information needs to be emphasized or suppressed.
- An improved Fast Iterative Deep Neural Network (FIDNN) is proposed based on parameter constraints and a momentum factor. Faster convergence speed and shorter testing runtime are obtained without stronger criteria compared to identical iteration-based methods.

## 2 Related works

PnP approaches have the benefit of being incredibly convenient. Time costs of parameter adjustment are better controlled by deep unfolding networks. We provide a brief review of two methods based on effective DCNN denoisers.

### 2.1 Plug-and-Play method

PnP methods have recently made significantly empirical progress, particularly with incorporation of learning-based denoisers. Moreover, Convolutional Neural Networks (CNN) have shown good performances through end-to-end training, e.g., FFDNet [10],TNRD [11] and DnCNN [23] for image denoising, DPDNN [19] and IRCNN [24] for non-blind deblurring. These methods demonstrate that CNN can train an excellent mapping function from a large number of degraded images to clean images. As a result, PnP approaches can make use of a pre-trained CNN denoiser to solve the Gaussian-like denoising subproblem

$$\begin{array}{c} x=\text{arg}\;\underset{x}{\text{min}}\;\{\frac{1}{2}{\left\|Hx-y\right\|}_{2}^{2}+\lambda \Phi \left(x\right)\}, \end{array}$$ (2)

where λ is a penalty parameter. PnP methods through variable splitting algorithms, such as HQS and SB, decouple data term and prior term of Eq (2). When HQS introduces an auxiliary variable *s*, Eq (2) becomes a constrained optimization problem given by

$$\begin{array}{c} \left(x,s\right)=\text{arg}\;\underset{x,s}{\text{min}}\;\{\frac{1}{2}{\left\|Hx-y\right\|}_{2}^{2}+\lambda \Phi \left(s\right)\},\;\text{s.t.}\;x=s. \end{array}$$ (3)

An equally constrained problem transforms into an unconstrained problem, namely

$$\begin{array}{c} \left(x,s\right)=\text{arg}\;\underset{x,s}{\text{min}}\;\{\frac{1}{2}{\left\|Hx-y\right\|}_{2}^{2}+\lambda \Phi \left(s\right)+\frac{\mu }{2}{\left\|x-s\right\|}_{2}^{2}\}, \end{array}$$ (4)

where *μ* denotes a penalty parameter. Above problem can be addressed by resolving iteratively following subproblems for *x* and *s* while holding remaining variables fixed,

$$\{\begin{array}{ll} x_{k}=\text{arg}\;\underset{x}{\text{min}}\;\{\frac{1}{2}\|Hx-y{\|}_{2}^{2}+\frac{\mu }{2}{\left\|x-s_{k-1}\right\|}_{2}^{2}\}, & \left(\text{5a}\right)\\ s_{k}=\text{arg}\;\underset{s}{\text{min}}\;\{\frac{1}{2{\left(\sqrt{\lambda /\mu }\right)}^{2}}{\left\|s-x_{k}\right\|}_{2}^{2}+\Phi (s)\}. & \left(\text{5b}\right) \end{array}$$

In this paper, *k* is iteration index. Eq (5a) has a closed-form analytic solution *x*,

$$\begin{array}{c} x_{k}=F^{-1}(\frac{\bar{F(H)}F\left(y\right)+\mu_{k}F(s_{k-1})}{\bar{F(H)}F\left(H\right)+\mu_{k}}), \end{array}$$ (6)

where the F(·), $F^{-1}\left(\cdot \right)$, and $\bar{F(H)}$ express FFT, inverse FFT, and complex conjugate of F(·), respectively. Gradient descent can also solve x-subproblem of Eq (5a) [19]. Any advanced Gaussian denoiser can be plugged into alternating iterations to solve z-subproblem. Therefore, numerous ill-posed inverse problems are quickly addressed using PnP approaches.

**Algorithm 1** Two-step iterative algorithm

**Initialization**:

 (1) Set $H,\bar{H},\gamma >0,k=1$;

 (2) Initialize *z*_0_, *x*_0_ = *y*.

**While** not converge **do**

 (1) Compute $z_{k}=x_{k-1}-\gamma_{k}\bar{H}(Hx_{k-1}-y)$;

 (2) Compute $x_{k}=f\left(z_{k},\sqrt{\lambda /\mu_{k}}\right)$;

 (3) *k* = *k* + 1.

**End while**

**Output**: *x*_*k*_

### 2.2 Deep unfolding network

Deep unfolding networks enhance interpretability of network structures in contrast to pure neural networks. Chen and Pock [11] proposed a flexible frame with a dynamic nonlinear diffusion model based on denoising tasks. Zhang and Ghanem [25] achieved proximal mapping related to sparsity-inducing regularizer without handcraft parameter adjustment. Tolooshams et al. [26] utilized an unfolding autoencoder neural network with an accelerated proximal gradient to learn compression matrix. Based on prior knowledge, model-based iterative networks with stationary layers are interpreted as the convolution and activation operations.

DCNN denoisers can be plugged into end-to-end deep unfolding networks to gain self-learning parameters. Wei et al. [22] achieved parameter automatic learning by proximal algorithms. Zheng et al. [27] used Hybrid ISTA to unfold ISTA with trainable parameters drawing in free-form Deep Neural Networks (DNN) to obtain guaranteed convergence. Jiu and Pustelnik [28] used primal-dual proximal iteration associated with standard penalized co-log-likelihood minimization to design a deep neural network. Iterative-based unfolding networks are used to achieve effectiveness of machine learning and adaptability of formula derivation.

## 3 Proximal gradient descent algorithm

### 3.1 Two-step iterative algorithm

Since deep unfolding networks are well-studied, it is interesting to integrate different degraded operations into an iterative algorithm. Different image restoration problems can be solved by studying uniformity of different degradation operations. To achieve this, a proximal operator is used to implement proximal gradient descent algorithm without splitting algorithms. Taylor expansion linearization equation [29] is calculated as

$$\begin{array}{c} x_{k}=\text{arg}\;\underset{x}{\text{min}}\;\{\begin{array}{c} \frac{1}{2}{\left\|Hx_{k-1}-y\right\|}_{2}^{2}+\frac{\mu }{2}{\left\|x-x_{k-1}\right\|}_{2}^{2}\\ +\langle \bar{H}(Hx_{k-1}-y),(x-x_{k-1})\rangle +\lambda \Phi \left(x\right) \end{array}\}, \end{array}$$ (7)

where *μ* denotes the penalty parameter, ${\left\|(x-x_{k-1})\right\|}_{2}^{2}$ denotes a proximal operator, *y* denotes degraded inputs, *x* denotes restored outputs. For image deblurring, $\bar{H}$ is a transpose convolution matrix. And by omitting a data term that is irrelevant to results, Eq (6) is merged into

$$\begin{array}{c} x_{k}=\text{arg}\;\underset{x}{\text{min}}\;\{\frac{\mu }{2}{\left\|x-x_{k-1}+\frac{1}{\mu }(\bar{H}(Hx_{k-1}-y))\right\|}_{2}^{2}+\lambda \Phi \left(x\right)\}. \end{array}$$ (8)

For the convenience of calculation, auxiliary variable *z* is introduced to substitute for complex and lengthy variable. Variable *z* is equal to

$$\begin{array}{c} z_{k}=x_{k-1}-\gamma_{k}\bar{H}(Hx_{k-1}-y), \end{array}$$ (9)

where *γ* is step size. Therefore, the solution can be expressed as

$$\begin{array}{c} x_{k}=\text{arg}\;\underset{x}{\text{min}}\;\{\frac{1}{2{\left(\sqrt{\lambda /\mu }\right)}^{2}}{\left\|x-z_{k}\right\|}_{2}^{2}+\Phi \left(x\right)\}. \end{array}$$ (10)

This is a Gaussian denoising problem with a standard deviation parameter $\sigma_{k}=\sqrt{\lambda /\mu_{k}}$. Clean images are gained using any existing DCNN denoiser, i.e., *x*_*k*_ = *f*(*z*_*k*_), where *f*(⋅) denotes a high-performing denoiser approximating a mapping equation. In summary, proposed iterative algorithm is summed up in **Algorithm 1**. The two-step algorithm is unfolded into an end-to-end neural network based on DCNN denoisers.

**Algorithm 2** Three-step iterative algorithm

**Initialization**:

 (1) Set $H,\bar{H},\gamma >0,\rho \in \left[0,1\right],k=1$;

 (2) Initialize $\bar{z_{0}},x_{0},b_{0}=y$.

**While** not converge **do**

 (1) Compute $\bar{z_{k}}=b_{k}-\gamma_{k}\bar{H}(Hb_{k}-y)$;

 (2) Compute $x_{k}=f\left(\bar{z_{k}},\sqrt{\lambda /\mu_{k}}\right)$;

 (3) Compute *b*_*k* + 1_ = *x*_*k*_ + *ρ*_*k*_(*x*_*k*_ − *x*_*k*−1_);

 (4) *k* = *k* + 1.

**End while**

**Output**: *x*_*k*_

### 3.2 Three-step iterative algorithm

#### 3.2.1 Fast iterative algorithm

Fast algorithms, e.g., Fast ADMM [30] and FISTA [31], show that convergence speed is accelerated by momentum factors. In this paper, we therefore adopt momentum factors to speed up convergence. Based on **Algorithm 1**, a momentum factor *ρ* is introduced to force the variable *x* to continue being calculated with a similar inertial force. The momentum factor falls between 0-1. The updated value of variable *x* is gotten by multiplying difference between two previous iterations by a momentum factor, i.e., *ρ*_*k*_(*x*_*k*_ − *x*_*k*−1_). A new variable *b* is equal to

$$\begin{array}{c} b_{k+1}=x_{k}+\rho_{k}(x_{k}-x_{k-1}). \end{array}$$ (11)

The new auxiliary variable $\bar{z}$ of accelerated methods changes due to the momentum factor *ρ*. Auxiliary variable becomes

$$\begin{array}{c} \bar{z_{k}}=b_{k}-\gamma_{k}\bar{H}(Hb_{k}-y), \end{array}$$ (12)

where *b* and *z* are intermediate variables of final results, *γ* represents step size. The fast iterative algorithm is summarized as **Algorithm 2**. Stimulated by IDNN, **Algorithm 2** can also be unfolded into a fast iterative deep neural network. Proposed fast method can accelerate convergence effectively, and detailed description will be given in Section 5.4. Moreover, FIDNN indeed shortens testing runtime than IDNN.

#### 3.2.2 Parameter constraint

Parameters including step size and a momentum factor are likely to affect image reconstructed solutions. The discovery that FIDNN might result in non-positive step size and momentum factors is in conflict with how these parameters are defined. To ensure positive convergence, these parameters including ${\left\{\gamma_{k},\rho_{k}\right\}}_{k=1}^{K=6}$ must also be subject to specific constraints. Parameter constraints [32] are guaranteed using auxiliary variables. These parameters follow a pattern in our implementation, in which *γ* smoothly decays with iterations, while *ρ* monotonously increases. With above rules, parameter constraint can be described as

$$\begin{array}{c} \left\{ \begin{array}{cc} \gamma_{k}=sp(w_{1}k+c_{1}), & w_{1}<0\\ \rho_{k}=\frac{sp(w_{2}k+c_{2})-sp(w_{2}+c_{2})}{sp(w_{2}k+c_{2})}, & w_{2}>0 \end{array}\right. \end{array}$$ (13)

where *sp*(*x*) is Softplus equation, i.e., *sp*(*x*) = ln(1+ exp(*x*)). The process that image restoration accords with meaning of model-based iterative solutions can be validly guaranteed.

## 4 Iterative deep neural networks

### 4.1 Deep unfolding network framework

**Fig 1:**
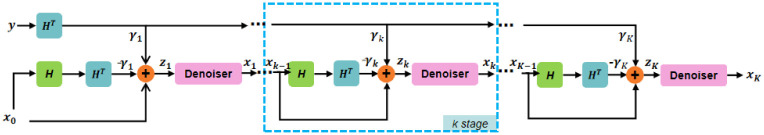
Framework of proposed iterative deep neural network.

**Algorithm 1** and **Algorithm 2** are unrolled into end-to-end iterative deep neural networks without numerous manual parameters. Network framework of **Algorithm 1** is shown in Fig 1. Model framework of **Algorithm 2** possesses a similar structure. One stage of proposed networks corresponds to one iteration of **Algorithm 1**. For *K* iterations, briefly introduce the first stage of forwarding propagation. First, variable $y\in R^{n_{y}}$ is equal to degraded inputs. Variables of *x*_0_ and *z*_0_ are initialized to variable *y*. Variables of *x*_0_ and *y* times downgraded operations. Add *x*_0_ to previous results to obtain *z*_1_. The *z*_1_ is processed by any efficient DCNN denoisers to get *x*_1_. Denoiser in this paper is high-performance U-Net [33]. The same procedures are carried out six times.

### 4.2 Deep convolutional neural network

**Fig 2:**
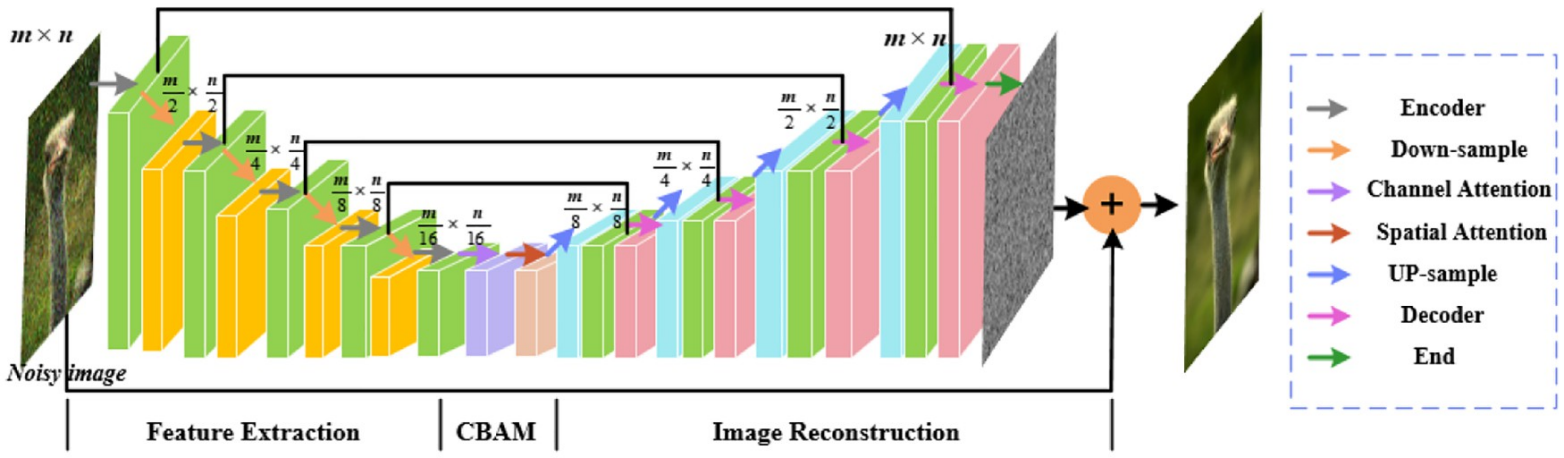
Model architecture of proposed DCNN denoiser. Right part is specific operations.

Pre-trained DCNN models are attractive to be used as denoisers. Zhang et al. [13] leveraged noise level maps as inputs to train denoiser for image restoration tasks. Tirer and Giryes [34] used IRCNN denoiser to solve image inpainting and deblurring problems. Li and Wu [35] exploited DnCNN denoiser to resolve depth image tasks. Romano et al. [36] utilized explicit regularization by pre-trained TNRD as a Gaussian denoiser to solve deblurring and super-resolution problems. Motivated by U-net for image segmentation, proposed U-net with only convolutional and activation operations is convenient to process for any size of natural images. Different from denoiser sub-network [19], our proposed methods introduce the attention mechanism to obtain attention mapping making up for inadequate weight information of image pixels. The proposed network contains three parts: feature extraction, Convolutional Block Attention Module (CBAM) [37] and image reconstruction, as shown in Fig 2.

In feature extraction module, there are four similar blocks. For each encoder layer, it consists of a convolution operation of 3×3 kernel and activation operations of Rectified Linear Unit (ReLU) nonlinearity to produce 64-channel feature maps. Each down-sample layer contains a convolution operation followed by an activation function. Receptive field is increased in down-sample layers to reduce spatial resolution of feature maps. Finally, there is an encoder layer with only a convolution and an activation operation. It is emphasized that feature maps are scaled twice as small by scaling factor 2 in down-sample layer, but image feature size is unchanged in encoder layer.

CBAM can be seamlessly integrated into any CNN architecture and trained by end-to-end methods together with basic CNN on account of CBAM is a lightweight general-purpose module. Attention maps are gained by sequentially computing two independent dimensions, namely channel, and space. Input feature maps are multiplied by attention maps to obtain adaptive feature refinement. Feature-channel relationship is exploited by channel attention to produce a channel attention map, focusing on “what” makes sense given an input. Spatial connections of image features are exploited by spatial attention to generate spatial attention maps, focusing on “where” is an informative element. Spatial attention is complementary to channel attention. Attention module effectively boosts information flow to learn which image information to be emphasized or suppressed. Comparative experiments on deblurring and denoising are done to demonstrate benefits of attention mechanism, as indicated in Section 5.2.

The image reconstruction module comprises up-sample layers that increase spatial resolution of feature maps followed by feature decoder layer. For each up-sample layer, it contains a transpose convolution operation of 3 × 3 kernel and ReLU nonlinearity to produce 64-channel feature maps. Feature maps are scaled twice as large by scaling factor 2 in up-sample layer. Reconstructed images suffer from a loss of some of their spatial information during feature extraction process. To compensate for loss of spatial information, cascaded feature maps are obtained by fusing one generated in up-sample layer with one generated in encoder layer. Cascading operations double the number of channels from 64 to 128. For decoder layers, there are five convolution layers. The first four have a convolution layer and ReLU nonlinearity. Only convolutional operations are used in final one. But feature map channel is adjusted through the first convolution operations from 128 to 64. The others generate 64-channel feature maps. Then feature maps are put into the last convolutional layer to generate the same number of channels as observed images. However, denoiser networks predict residual parts instead of directly utilizing outputs of the last convolutional layer as reconstructed images, which has been proved to be more robust. Therefore, a shortcut is exploited from inputs to reconstructed images.

## 5 Experiments and results

### 5.1 Training process

#### 5.1.1 Training dataset

Observed images are gained utilizing different degraded operations. For denoising, clean images are added with AWGN for different noise levels to produce noisy images. For deblurring, blurry images are gained by convolving clear images with different blur kernels and adding AWGN. Training dataset is DIVerse 2K (DIV2K) resolution image dataset [38]. Each image is randomly cropped into 1000 images of size 128. During training process, these inputs are cropped into 64 size patches. To realize data augmentation, cropped randomly patches are flipped and rotated to generate a total of 250,000 ones.

#### 5.1.2 End-to-end training

Each DCNN denoisers shares the same parameters to reduce numerous parameters and prevent overfitting. In our implementation, networks are trained using Mean Square Error (MSE) loss function

$$\begin{array}{c} \Theta =\text{arg}\;\underset{\Theta }{\text{min}}\{\sum_{n=1}^{N}{\left\|F(y_{n};\Theta )-x_{n}\right\|}^{2}\}, \end{array}$$ (14)

where *y*_*n*_ and *x*_*n*_ are *i*th pair of damaged and clear image patches, $F(y_{n};\Theta )$ is proposed networks with parameters Θ. ADAM optimizer [39] is utilized to optimize parameters. Convolutional kernels are initialized by Xavier initializers developed in [40]. Warmup scheduler strategy is adopted for learning rate. Learning rate remains constant over the first three epochs of early training. We in later epochs use CosineAnnealingLR strategy. Learning rate is initially set to 0.0002. Proposed network is implemented under Pytorch framework and trained by Nvidia RTX 3090. Denoising experiment takes about 32 hours to achieve convergence, while deblurring experiment takes about 48 hours. Parameter *γ* initialization of IDNN is set to 1.0. Parameters {*w*_1_, *c*_1_, *w*_2_, *c*_2_} of FIDNN are initialized as {−0.2, 0.5, 1.2, 0.0}.

### 5.2 Ablation study

Regarding the effects of attention module with U-Net, we conduct whether models have CBAM or not. Several comparative experiments are in Tables 1 and 2. DCNN_N represents a DCNN denoiser without CBAM. FIDNN_N represents a fast iterative network without CBAM. When noise level is high, DCNN denoiser with attention mechanism makes great progress. Regarding Gaussian deblurring experiments from Table 2, FIDNN increases PSNR value by 0.25. Therefore, information flow is effectively taught which information needs to be emphasized or suppressed due to attention module. In future trials, methods proposed in this paper are all introduced into attention mechanism.

**Table 1:** Ablation study of denoising on Set12 dataset.

Methods	DCNN_N	DCNN	FIDNN_N	FIDNN
Noise level 15	32.82	32.83	32.89	**32.91**
Noise level 25	30.49	30.52	30.56	**32.58**
Noise level 50	27.34	27.39	27.47	**27.53**

**Table 2:** Ablation study of deblurring on Set10 dataset.

Methods	DCNN_N	DCNN	FIDNN_N	FIDNN
Gaussian blur for noise level 2				
Standard deviation 1.2	33.51	33.65	33.60	**33.85**
Standard deviation 1.6	30.53	30.63	30.91	**31.13**
Motion blur for noise level 7.65				
Levin 19×19 [45]	28.90	28.98	29.12	**29.17**
Levin 17×17 [45]	28.49	28.55	28.56	**28.66**

To verify the effectiveness of consolidation degradation operations, we implement two types of experiments, i.e., DCNN denoisers and iterative network FIDNN. Comparable trials are shown in Tables 1 and 2. For denoising and deblurring, FIDNN without attention mechanism improves maximum PSNR gains by up to 0.13 and 0.38, respectively. The 0.5 and 0.19 gains of average PSNR in gaussian and motion deblurring are realized over pure denoisers with CBAM, demonstrating the significance of integrating degradation operations into unfolding networks.

### 5.3 Image restoration results

#### 5.3.1 Denoising

We compare our methods with several state-of-the-art denoising methods, including two model-based methods, i.e., BM3D [41] and EPLL [42], and three learning-based methods, i.e., TNRD, DPDNN, and IRCNN. Average PSNR results of different methods are shown in Table 3 on widely-used Set12 dataset [23]. Learning-based methods are superior to model-based methods. DPDNN greatly outperforms IRCNN and TNRD, while FIDNN performs better for higher noise levels than DPDNN. We also test denoising results of Color Berkeley Segmentation Dataset (CBSD68) [43] and Kodak24 dataset [44], as shown in Table 4. Model-based method, i.e., CBM3D [41], is outperformed by FIDNN to 0.81 average PSNR gains for noise level 50 on CBSD68 dataset.

**Table 3:** PSNR results of denoising by different methods on Set12 dataset.

Image	C.man	House	Peppers	Starfish	Monor	Airpl	Parrot	Lena	Barbara	Boat	Man	Couple	Average
Noise level 15													
BM3D [41]	31.93	34.94	32.70	31.16	31.86	31.08	31.38	34.27	33.11	32.14	31.93	32.12	32.39
EPLL [42]	31.81	34.13	32.58	31.07	32.03	31.16	31.41	33.86	31.33	31.92	31.97	31.89	32.10
TNRD [11]	32.15	34.56	33.02	31.76	32.55	31.45	31.65	34.25	32.15	32.13	32.25	32.08	32.50
IRCNN [24]	32.53	34.88	33.21	31.96	32.98	31.66	31.88	34.50	32.41	32.36	32.36	32.37	32.76
DPDNN [19]	32.44	**35.40**	33.19	32.06	**33.32**	**31.78**	31.45	**34.80**	**32.81**	**32.55**	**32.52**	32.51	32.90
IDNN	32.54	35.19	**33.38**	**32.23**	33.16	**31.78**	**31.99**	34.70	32.57	32.41	32.43	32.49	**32.91**
FIDNN	**32.57**	35.19	33.36	32.22	33.16	31.75	31.97	34.71	32.56	32.43	32.44	**32.53**	**32.91**
Noise level 25													
BM3D [41]	29.46	32.86	30.16	28.56	29.25	28.43	28.93	32.07	30.72	29.90	29.63	29.72	29.97
EPLL [42]	29.25	32.04	30.06	28.44	29.30	28.56	28.91	31.63	28.56	29.68	29.62	29.48	29.63
TNRD [11]	29.70	32.52	30.53	29.03	29.85	28.89	29.19	31.99	29.42	29.90	29.89	29.73	30.05
IRCNN [24]	30.12	33.02	30.81	29.21	30.20	29.05	29.47	32.40	29.93	30.17	30.02	30.05	30.37
DPDNN [19]	30.12	**33.55**	30.90	29.43	30.31	29.14	29.28	32.69	**30.30**	**30.34**	**30.15**	**30.24**	30.54
IDNN	**30.16**	33.47	**31.05**	**29.50**	**30.42**	29.21	29.55	**32.72**	30.02	30.26	30.10	30.21	30.56
FIDNN	**30.16**	33.52	30.99	29.47	**30.42**	**29.24**	**29.56**	**32.72**	30.04	30.27	30.13	30.20	**30.58**
Noise level 50													
BM3D [41]	26.14	29.69	26.68	25.03	25.82	25.11	25.90	29.04	27.23	26.79	26.82	26.46	26.73
EPLL [42]	26.02	28.75	26.62	25.05	25.79	25.24	25.83	28.44	24.80	26.66	26.73	26.22	26.35
TNRD [11]	26.61	29.46	27.13	25.42	26.30	25.60	26.09	28.96	25.71	26.95	27.00	26.50	26.82
IRCNN [24]	27.16	29.90	27.33	25.48	26.66	25.78	26.48	29.36	26.17	27.17	27.14	26.86	27.12
DPDNN [19]	27.12	**31.04**	27.44	25.95	27.00	25.97	26.47	29.86	**27.22**	**27.42**	27.32	27.23	27.50
IDNN	27.25	30.76	27.52	25.75	26.95	**26.05**	26.59	29.79	26.50	27.38	27.28	27.19	27.42
FIDNN	**27.29**	30.80	**27.74**	**25.96**	**27.12**	26.02	**26.67**	**29.87**	26.88	27.37	**27.35**	**27.28**	**27.53**

**Table 4:** Average PSNR results of denoising by different methods on CBSD68 and Kodak24 datasets.

Datasets	Noise level	CBM3D [41]	IRCNN [24]	DnCNN [23]	FFDNet [10]	DPDNN [19]	FIDNN
CBSD68	15	33.47	33.87	33.89	33.88	33.99	**34.02**
	25	30.69	31.18	31.23	31.22	31.30	**31.35**
	50	27.37	27.88	27.92	27.97	28.14	**28.18**
Kodak24	15	34.41	34.69	34.59	34.63	34.73	**34.80**
	25	31.81	32.15	32.13	32.13	32.12	**32.21**
	50	28.62	28.94	28.95	29.11	29.11	**29.23**

**Fig 3:**
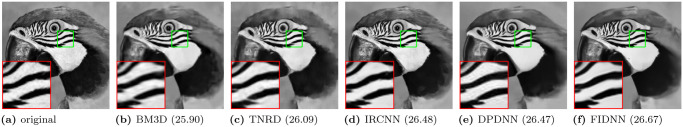
Gray image denoising results for noise level 50 on ‘Parrot’ image from Set12 dataset. (a) original. (b) BM3D (25.90). (c) TNRD (26.09). (d) IRCNN (26.48). (e) DPDNN (26.47). (f) FIDNN (26.67).

**Fig 4:**
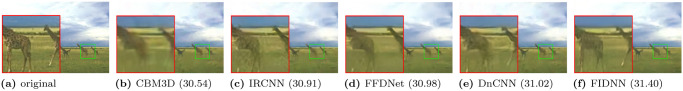
Color image denoising results for noise level 50 on ‘253055’ image from CBSD68 dataset. (a) original. (b) CBM3D (30.54). (c) IRCNN (30.91). (d) FFDNet (30.98). (e) DnCNN (31.02). (f) FIDNN (31.40).

**Fig 5:**
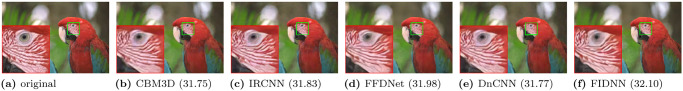
Color image denoising results for noise level 50 on ‘Kodim23’ image from Kodak24 dataset. (a) original. (b) CBM3D (31.75). (c) IRCNN (31.83). (d) FFDNet (31.98). (e) DnCNN (31.77). (f) FIDNN (32.10).

Qualitative results of gray images for noise level 50 are shown in Fig 3. DPDNN is surrounded by edge connections, while FIDNN is filled with better and smooth image details. Visual effects of color images are shown in Figs 4 and 5. CBM3D is too smooth to preserve the edge. Three learning-based methods suffer from poor edge preservation of small objects at a distance. In contrast, proposed method benefits from comprehensive textures and sharper edges.

#### 5.3.2 Deblurring

Deblurring experiments of non-linear blur kernels are carried out to further confirm wide applicability of proposed methods, as shown in Tables 5 and 6. The blur kernel includes Gaussian blur of size 25 with standard deviations of 1.2 and 1.6, and motion blur of size 19 and 17 in [45]. For Gaussian deblurring, AWGN for noise level 2 is added to blurred images. For motion deblurring, add AWGN for noise level 7.65 to them. The model-based method, i.e., IDDBM3D [46], and four learning-based methods, including IRCNN, IRCNN+ [13], DPIR [13], and DPDNN are compared with our methods on widely-used Set10 dataset [19]. IRCNN+ refers to the method [13] in which the denoiser sub-network is replaced with IRCNN. Model-based methods perform poorly while processing Gaussian blur. Compared to the same iteration-based method, i.e., DPDNN, 0.12 gains of average PSNR for motion blur of size 17 are acquired by FIDNN. In color image dataset, FIDNN outclasses two PnP approaches.

**Table 5:** PSNR results of deblurring by different methods on Set10 dataset.

Image	Barbara	Boats	Butterfly	C.Man	House	Leaves	Lena	Parrot	Peppers	Starfish	Average
Gaussian blur with standard deviation 1.2 for noise level 2											
IDDBM3D [46]	31.92	33.33	32.17	30.18	35.60	33.18	33.12	34.55	31.74	32.90	32.60
IRCNN [24]	31.40	33.40	32.45	30.44	35.51	33.68	33.44	34.58	32.00	33.42	33.03
IRCNN+ [13]	31.32	32.69	32.45	30.04	34.40	33.13	32.80	33.83	31.49	32.72	32.49
DPDNN [19]	31.62	33.81	33.33	30.84	36.01	34.01	34.13	35.51	32.19	34.23	33.57
IDNN	31.60	33.86	33.20	30.83	35.94	34.15	34.14	35.51	32.35	34.33	33.59
FIDNN	**32.39**	**34.02**	**33.40**	**30.93**	**36.16**	**34.39**	**34.38**	**35.72**	**32.44**	**34.62**	**33.85**
Gaussian blur with standard deviation 1.6 for noise level 2											
IDDBM3D [46]	25.99	31.17	29.79	27.68	33.56	30.13	30.91	31.90	29.64	30.57	30.13
IRCNN [24]	26.15	31.41	30.44	28.06	33.79	30.43	31.14	31.82	30.68	30.77	30.47
IRCNN+ [13]	25.77	30.87	30.06	27.65	32.81	30.14	30.83	31.64	29.79	30.42	30.00
DPDNN [19]	**26.47**	31.54	30.67	28.24	**34.25**	30.23	31.48	32.40	30.18	32.00	30.75
IDNN	25.60	31.62	31.13	28.63	33.96	30.95	31.65	32.79	30.90	31.77	30.90
FIDNN	26.02	**31.80**	**31.42**	**28.84**	34.20	**31.48**	**31.79**	**32.90**	**30.98**	**32.12**	**31.13**
19 × 19 motion blur kernel 1 of [38] for noise level 7.65											
IRCNN [24]	28.18	29.12	**28.51**	28.11	32.03	**28.41**	29.51	31.07	**28.87**	27.86	**29.17**
IRCNN+ [13]	28.29	29.03	27.99	**28.31**	31.70	27.73	29.56	30.74	28.68	27.55	28.96
DPDNN [19]	28.01	29.19	28.24	27.77	**32.06**	27.98	29.42	31.03	28.42	**28.00**	29.01
IDNN	28.14	**29.36**	28.23	27.89	**32.06**	27.81	29.70	31.17	28.79	27.87	29.10
FIDNN	**28.22**	29.33	28.30	28.06	**32.06**	27.96	**29.77**	**31.23**	28.76	27.99	**29.17**
17 × 17 motion blur kernel 2 of [38] for noise level 7.65											
IRCNN [24]	**27.36**	28.94	**28.20**	27.70	**31.94**	**27.91**	29.27	30.67	**28.71**	27.67	**28.84**
IRCNN+ [13]	27.34	28.78	27.77	**27.76**	31.42	27.38	29.17	30.37	28.36	27.46	28.58
DPDNN [19]	26.86	28.84	27.47	27.48	31.91	27.28	29.23	30.46	28.02	**27.82**	28.54
IDNN	26.63	28.82	27.37	27.25	31.69	26.86	29.30	30.60	28.28	27.34	28.41
FIDNN	26.76	**29.06**	27.60	27.40	31.91	27.48	**29.46**	**30.81**	28.47	27.64	28.66

**Table 6:** Average PSNR results of Gaussian deblurring for different standard deviation by different methods on Kodak24 dataset.

Methods	IRCNN [24]	IRCNN+ [13]	DPDNN [19]	DPIR [13]	FIDNN
Standard deviation 1.2	32.96	32.17	32.89	32.69	**32.99**
Standard deviation 1.6	30.40	29.70	30.46	30.03	**30.55**

**Fig 6:**
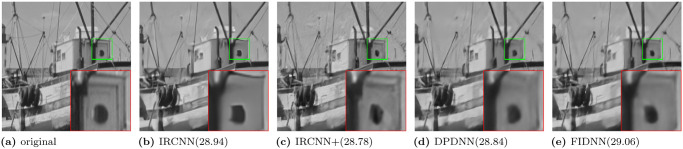
Motion deblurring results for kernel size 17 and noise level 7.65 on ‘Boat’ image from Set10 dataset. (a) original. (b) IRCNN(28.94). (c) IRCNN+(28.78). (d) DPDNN(28.84). (e) FIDNN(29.06).

**Fig 7:**
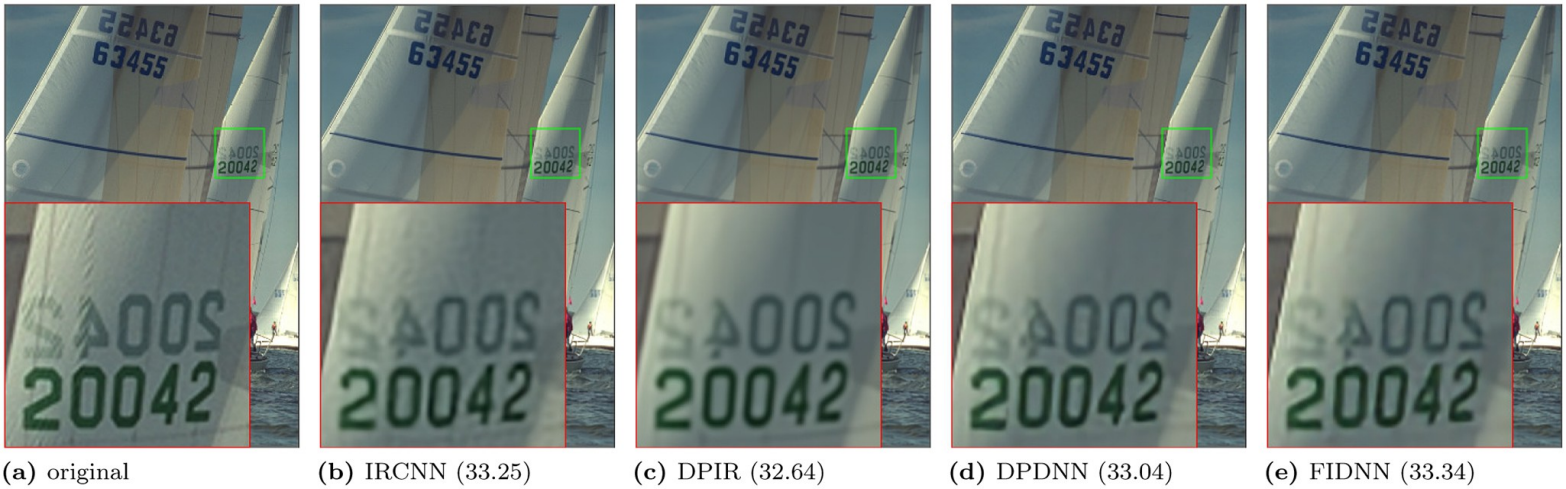
Gaussian deblurring results for standard deviation 1.6 and noise level 2 on ‘kodim19’ image from Kodak24 dataset. (a) original. (b) IRCNN (33.25). (c) DPIR (32.64). (d) DPDNN (33.04). (e) FIDNN (33.34).

Qualitative results of deblurring experiments are shown in Figs 6 and 7. IRCNN is so smooth that it produces distorted edges. IRCNN+ and DPDNN are encircled by obvious motion artifacts. However, FIDNN effectively defeats motion artifacts as well as enjoys sharper edges and a more pleasant texture structure than other methods.

### 5.4 Convergence analysis

**Fig 8:**
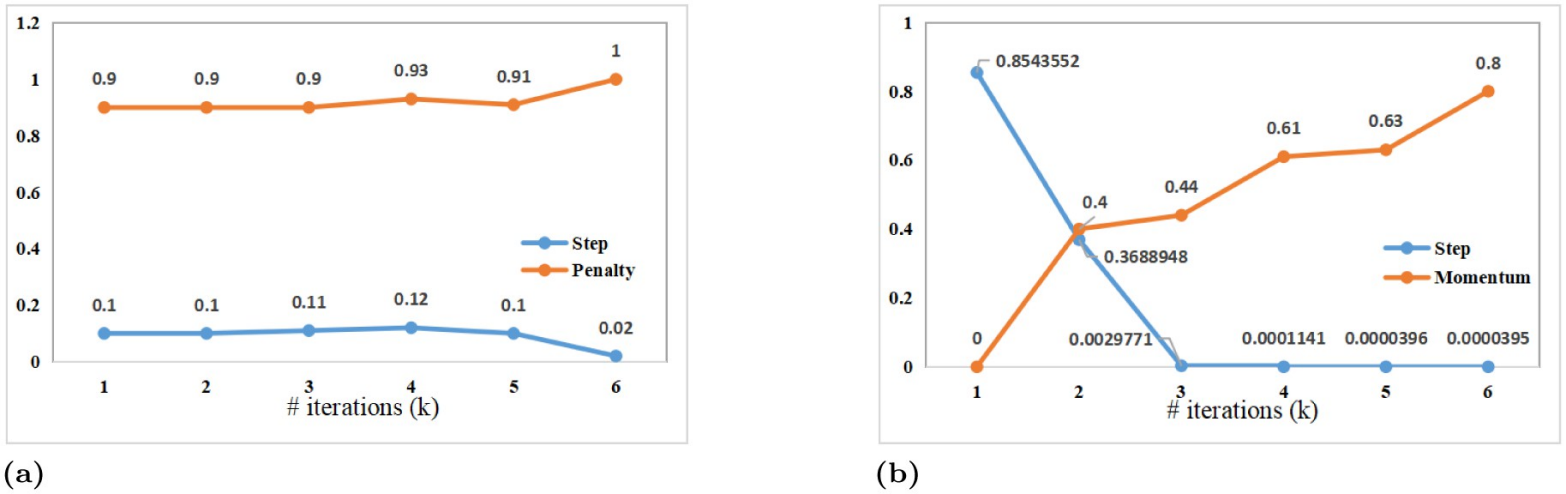
Parameter variations on motion deblurring with kernel size 19. (a): step size and penalty parameter of DPDNN; (b): step size and momentum parameter of FIDNN.

**Fig 9:**
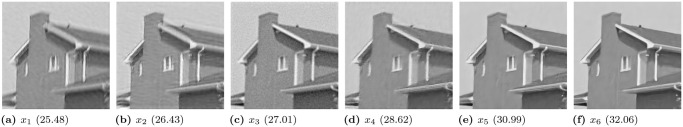
Gray deblurring results and parameter variations on each iteration for motion blur with kernel size 19 on ‘House’ image from Set10 dataset. (a) x1 (25.48). (b) x2 (26.43). (c) x3 (27.01). (d) x4 (28.62). (e) x5 (30.99). (f) x6 (32.06).

Effects of detail restoration are likely to be affected by the trend of parameter variation. Under the same configuration, step size of DPDNN shows a downward trend, and penalty parameter shows an upward trend from Fig 8a. This is consistent with meaning of parameters mentioned in this paper. However, DPDNN in later iterations shows a very modest fluctuation. Its unstable noise variance and blur composition go counter to iterative solutions. In early stages, parameters of FIDNN change rapidly. Correspondingly, degraded images become clearer quickly, as shown in Fig 9. Therefore, parameter variation provides a clearer explanation of what an iterative solution means.

**Fig 10:**
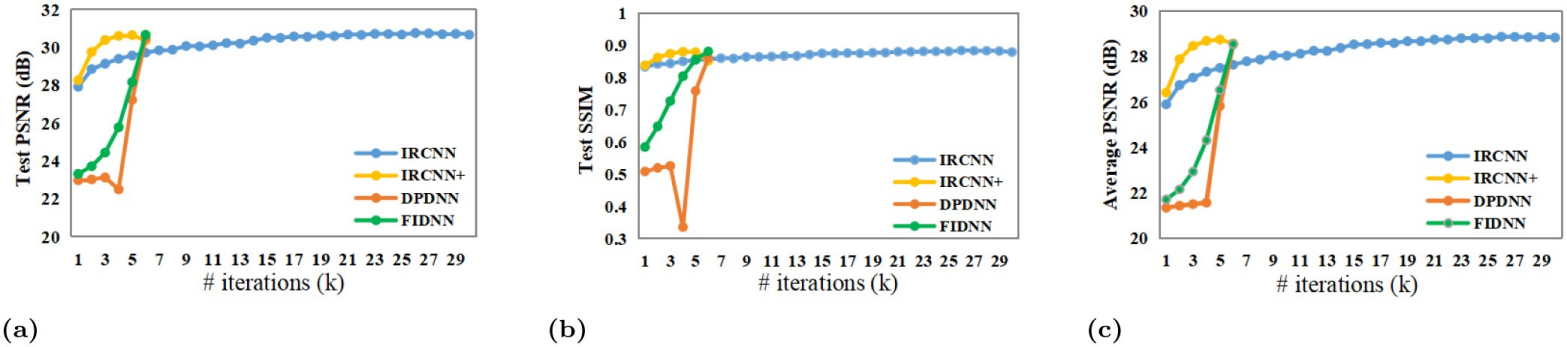
Comparison of PSNR and SSIM results for motion blur kernel size 17 and noise level 7.65. (a): PSNR results on ‘Parrrot’ image; (b): SSIM results on ‘Parrrot’ image; (c): Average PSNR results on Set10 dataset.

Quantitative experiments are shown in Fig 10 to demonstrate influence of parameters. Under the same configuration, DPDNN yields vital fluctuations in intermediate periods. It may be connected to parameter instability. IRCNN sacrifices more iterations to achieve good-performing results. IRCNN+ converges quickly in early stages, but its stability is poor. Contrarily, FIDNN remains fast and stable convergence with a lower number of iterations.

**Table 7:** FLOPs (in G) and parameters on image size 256 × 256. Runtime (in seconds) of each image and average RSNR (in dB) for noise level 50 on Kodak24 dataset.

Methods	IRCNN [24]	DnCNN [23]	FFDNet [10]	DPDNN [19]	IDNN	FIDNN_N	FIDNN
FLOPs	12.180	37.112	15.903	149.430	149.431	149.430	149.431
Paras	185.857K	556.289k	485.316K	1.290M	1.364M	1.364M	1.364M
Run time	0.034	0.048	0.020	0.161	0.218	0.178	0.189
PSNR	28.94	28.95	28.98	29.11	29.15	29.19	29.24

The experiments are implemented in Pytorch framework on a PC with an Intel core i7-11700k CPU and an Nvidia RTX 3090 GPU.

### 5.5 Model complexity and runtime

Under the same hardware equipment, we test model complexity and testing runtime for several deep learning methods, as shown in Table 7. IRCNN gains the best performance in model FLOPs and parameters. With the same U-Net denoiser, FIDNN_N owns better results of average PSNR for higher noise levels than DPDNN. Numerous convolution parameters of FIDNN result in longer testing runtime. It is worthwhile to mention that runtime of FIDNN does decrease distinctly over IDNN per image.

## 6 Conclusion

This work links variational models of model-based methods to learnable deep learning approaches. Firstly, the proximal operator is used to implement Taylor expansion linearization under energy minimization of a variational function. Proximal gradient descent algorithm is unrolled to IDNN model with proposed U-Net denoiser by end-to-end training. The attention mechanism incorporated into denoiser sub-network effectively understands emphatic or suppressive image information. Furthermore, by introducing a momentum factor that drives reconstruction results to continue iterating with inertial force, IDNN is extended to fast IDNN (FIDNN) without stronger conditions to speed up the convergence.

Self-learning parameters in this paper through an end-to-end approach effectively reduce manually tuning costs. Moreover, proposed iterative solution with trainable parameters can express dynamic characteristics of image reconstruction than constant parameters. The experimental results show that FIDNN with fewer iterations has more stable and faster test convergence than several iterative-based unfolding methods. Due to extensive applicability of proposed models, more computer vision tasks in the future can be addressed by handling different degraded operations simultaneously.
